# Five ixodid tick species including two morphotypes of *Rhipicephalus turanicus* on nestlings of Eurasian eagle owl (*Bubo bubo*) from south-eastern Bulgaria

**DOI:** 10.1186/s13071-021-04832-0

**Published:** 2021-06-26

**Authors:** Attila D. Sándor, Boyan Milchev, Nóra Takács, Jenő Kontschán, Sándor Szekeres, Sándor Hornok

**Affiliations:** 1 Department of Parasitology and Parasitic Diseases, University of Agricultural Sciences and Veterinary Medicine of Cluj-Napoca, Calea Mănăştur 3-5, 400337 Cluj-Napoca, Romania; 2 Department of Parasitology and Zoology, University of Veterinary Medicine, Budapest, Hungary; 3 Wildlife Management Department, University of Forestry, Sofia, Bulgaria; 4 Plant Protection Institute, Centre for Agricultural Research, Budapest, Hungary

**Keywords:** Birds, Ixodidae, Parasitism, Habitat structure, Host–parasite relationship

## Abstract

**Background:**

Birds are major hosts for many tick species (Acari: Ixodidae, Argasidae), and their role is especially important in transporting ticks over large distances along their seasonal migratory routes. Accordingly, most studies across Europe focus on the importance of avian hosts in tick dispersal, and less emphasis is laid on resident birds and their role in supporting tick life cycles. Eurasian eagle owls (*Bubo bubo*) exemplify the latter, but all the few studies on their tick infestation were carried out in Western Europe and even those did not involve a large sample size and did not assess infestation prevalence in natural habitats.

**Methods:**

In this study, 320 ixodid ticks were collected from nestlings of Eurasian eagle owls during the period 2018–2020 in Bulgaria in south-eastern Europe. These ticks were analysed morphologically, and selected specimens molecularly based on cytochrome *c* oxidase subunit I (*cox*1) gene. The effects of environmental and habitat-related conditions and of the species of prey eaten by eagle owls on tick infestation were also evaluated.

**Results:**

The majority of ticks were identified as adults of *Rhipicephalus turanicus* (*n* = 296). In addition, 15 *Hyalomma marginatum* (three males, 11 nymphs and a larva), one female of *Haemaphysalis erinacei* and of *Ha. punctata*, and a nymph of *Ixodes ricinus* were found. Among *R. turanicus*, two distinct morphotypes were observed, but they do not form a monophyletic clade in the phylogenetic tree based on the mitochondrial gene cox1. We found a positive correlation between the total number of ticks on nestlings from a particular nest and the number of medium-sized to large prey mammals brought to the nestling owls. Also, the most important predictor for tick abundance was the effect of the extent of arable land (negative), while forests and grasslands contributed less, with no effect observed in case of urbanized areas and watercourses.

**Conclusions:**

The intensity of tick infestation can be high on nestling Eurasian eagle owls (mean intensity 16.59 ticks/nestling). In this study, five different tick species were recorded, among which *R. turanicus* dominated. Two male morphotypes of this tick species were found, but their morphological differences were not reflected by genetic diversity or phylogenetic clustering. The most important factor determining tick abundance was the land-use structure.

**Graphic Abstract:**

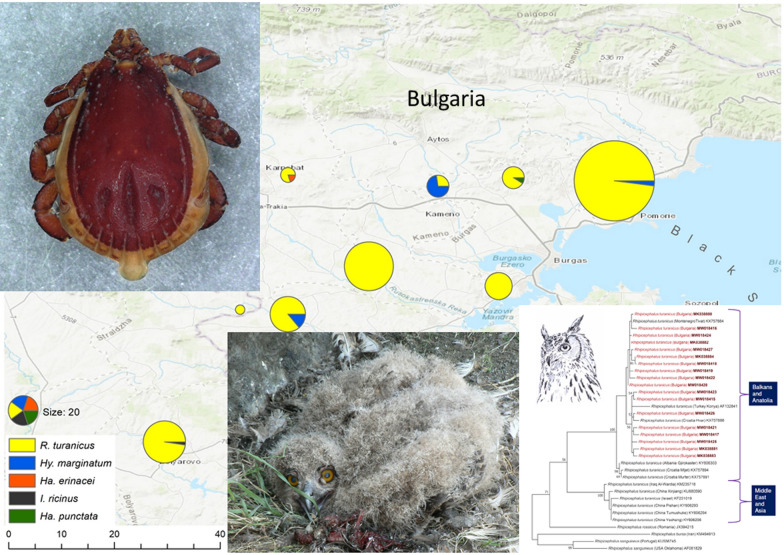

**Supplementary Information:**

The online version contains supplementary material available at 10.1186/s13071-021-04832-0.

## Background

Ticks (Acari: Ixodidae, Argasidae) are among the most widespread ectoparasites of terrestrial vertebrates, only surpassed in diversity by mesostigmatid mites [1]. All tick species are haematophagous, with at least one blood meal taken by each development stage (except for adult males of certain species, which do not feed). Through their blood feeding, ticks not only deplete the energetic resources of their respective host, but may be important vectors for pathogens of viral [2], bacterial or parasitic diseases worldwide [3]. In particular, ticks are among the most important vectors of zoonotic pathogens causing diseases in most temperate regions [4] and especially in Europe [5]. Among terrestrial vertebrates, birds are major hosts of several hard ticks (Ixodidae), and their role is especially important as transporters of engorged ticks over large distances along their seasonal migratory routes [6, 7]. Members of the genus *Ixodes* are the most common parasites of birds in Europe [8]; however, ticks from other genera may also infest birds [9–11]. Thus, migratory passerine birds caught in Europe frequently carry subadult stages of *Hyalomma* [12] and *Haemaphysalis* species [13, 14], while members of the genus *Rhipicephalus* have been observed on larger birds in south-western Europe [9, 10].

Most European studies investigating the relationship between ticks and avian hosts focus on ticks of migratory birds. However, not only migratory birds are important tick hosts, as resident species may serve as high-density hosts [15], especially in the case of bird species feeding or breeding on the ground [16]. Papers reporting tick parasitism of resident populations are usually targeting one or a few host species, with game birds [17] and seabirds [18] being the most common groups studied, but the ecology of bird-specialist ticks is also well documented in passerines [19, 20]. Owls (Strigiformes) are rarely studied as tick hosts, due to their scarcity, nocturnal habits or hard-to-access nesting sites [21]. The few studies of ectoparasites of owls are either surveys of nest material [22, 23] or anecdotal reports from rehabilitation centers of findings of injured birds [10]. In general, tick parasitism is considered rare in this group [24].

Eurasian eagle owl (*Bubo bubo*) is not an exception, with only four reports on their tick parasites. Fain et al. [22] lists a few individuals found in nest material, two studies report on the ticks collected from individuals brought to recovery centers [9, 10], while Ortego and Espada [25] reported the general impact of parasites and pathogens on nestling development. All studies on eagle-owl ticks were conducted in Western Europe, with no such information available in the eastern part of the continent. We failed to find any study targeting ticks of wild birds in Bulgaria; even in neighboring countries, all but one study [14] targeted migratory populations of birds [6, 26]. In addition, we found no study listing ticks of eagle owls either in Bulgaria [27] or in neighboring countries.

Eagle owls are the largest nocturnal predatory birds in Europe, breeding all over the continent. They do not build nests, and the species lays its eggs directly on the ground in a nest scrape (rarely in abandoned twig nests of other species). The nests are mostly found on rock ledges or larger crevices, or directly on the ground. Such nest sites are used for long periods, sometimes for many generations [21]. Several such breeding territories are surveyed in south-eastern Bulgaria on a yearly basis as part of an ongoing study of the species [28].

Our aim was to evaluate the tick parasitism of nestling eagle owls in south-eastern Europe (Bulgaria), providing details on species and developmental stages found on nestlings and to suggest possible sources for the interesting tick assemblages found.

## Methods

### Tick collection

**Fig. 1:**
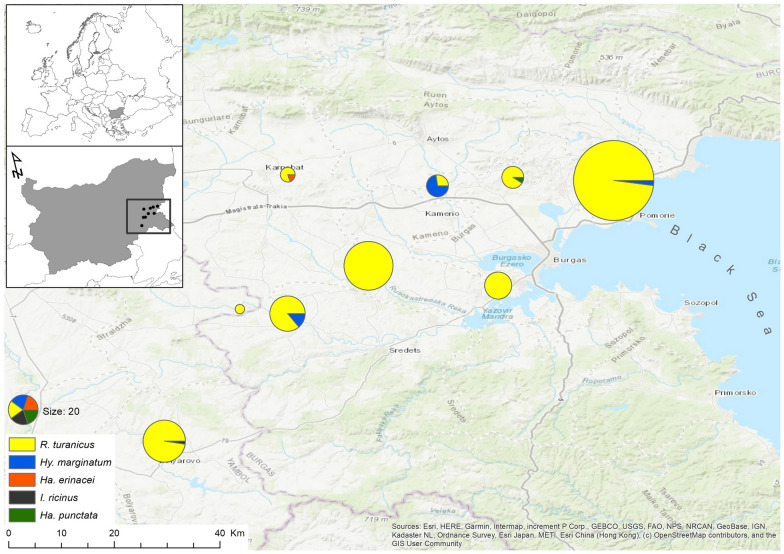
Map with the location of Eurasian eagle owl (*Bubo bubo*) nests used for tick sampling, with tick species and numbers recorded at each location

The ticks were collected from nestlings of Eurasian eagle owls in nests and their surroundings kept under surveillance in south-eastern Bulgaria (Burgas region) during the nesting seasons in 2018–2020 (May–June). Altogether, nine different breeding sites were monitored each year, with ticks collected at each location, but not in each year, due to low breeding success in the region [29]. As this species is subject to illegal destruction or poaching, we omitted the names and accurate coordinates of the nest locations (Fig. 1). Each nest was visited at least twice to evaluate breeding productivity and to collect prey remains in order to establish the resources used locally by eagle owls [28]. The data for identified prey species were also used to locate the putative host sources of ticks found on nestlings. At each visit, nestlings were inspected for ticks, especially on the head, the neck, the ears and on the underwing and anal zones. All ticks observed were collected with fine tweezers and stored in 97% ETOH in individual tubes, with different tubes used for each individual bird. All nest surveys and nestling manipulations were performed according to the wildlife monitoring protocol of the Wildlife Management Department, University of Forestry, Sofia, and in accordance with national legislation in Bulgaria.

### Morphological identification of ticks

Ticks were identified in a laboratory using morphological keys [30–32] and assigned to species, developmental stage and sex (only adults). Because ticks identified as *R. turanicus* showed significant variations in morphology (‘small’ and ‘large’ males), ten randomly selected individuals of each morphotype were used for morphological measurements and genetic characterization.

Measurements were performed with an Olympus BX61 microscope, using a DP72 digital camera equipped with Cell^F^ software (Olympus Corporation, Tokyo, Japan). To measure the different morphometric features of ticks, we followed Sándor et al. [33] (Additional file 1). In addition, the adanal plates were photographed and shapes defined according the selected landmark points (see details of landmark point selection in Bakkes et al. [34], Suppl. Fig. S1). The selected individuals were cleansed in water, placed directly on microscope slides, and covered with cover slips, without fixation. For clarification we used one drop of lactophenol, placed directly on the samples. Pictures of the same males were taken using a VHX-5000 (Keyence Co., Osaka, Japan) digital microscope, and were used here for the figures. Morphological measurements were compared using two-tailed Student’s *t* test; significance levels were set to *p* < 0.05.

### DNA extraction and phylogenetic analysis

The same tick individuals (10 males from each morphotype) and five randomly chosen ‘large’ individuals (to include individuals from all the different nest sites) were used for genetic characterization. After DNA extraction, an approximately 710-bp-long fragment of the cytochrome *c* oxidase subunit I (*cox*1) gene of the mitochondrial genome was amplified with the primers HCO2198 (5′-TAA ACT TCA GGG TGA CCA AAA AAT CA-3′) and LCO1490 (5′-GGT CAA CAA ATC ATA AAG ATA TTG G-3′) as reported [35]. Purification and sequencing was done by Biomi Ltd. (Gödöllő, Hungary). Obtained sequences were manually edited, then aligned and compared to those available in GenBank™ using the Basic Local Alignment Search Tool (BLAST, https://blast.ncbi.nlm.nih.gov). In the phylogenetic analyses reference sequences with high coverage (i.e. 99–100% of the region amplified here) were retrieved from GenBank and analysed. Phylogenetic analyses were conducted by MEGA version 7.0 using the Maximum-Likelihood method, Hasegawa–Kishino–Yano (HKY) model according to the selection of the program and 1000 bootstraps. Mean sequence divergences among the major clades were calculated using MEGA. Representative sequences (MK03880–MK03884, MW018415–MW018427) were submitted to GenBank.

### Environmental predictors used for modelling habitat-generated differences in tick distribution

To assess the causes of differences in tick parasitism between the different nests, we collected environmental and habitat-related information for each individual eagle owl nest and evaluated the prey selection of individual owl pairs using the prey remains collected from the nest sites. We built a multiple regression model for testing the relative contribution of different land use categories in predicting tick abundance. For independent variables we used the area of different land use categories (forest cover, grassland, arable land and urban areas within a 3 km radius of the nests’ proximity). We hypothesized that the different land-use categories (the small scale habitats therein) may predict the difference in tick faunas (caused by the differences in abundance of suitable hosts and microclimate conditions, see also [36]). We selected a 3 km radius circle, which covers an area of 28.2 km^2^, corresponding to the estimated breeding home range of individual Eurasian eagle owls [37]. The source for this dataset was the CORINE Land Cover database, 2016 version (no climate-related data were used). The dataset was provided by the European Environment Agency (EEA, http://www.eea.europa.eu/). All statistical differences were considered significant for *p* < 0.05.

## Results

Altogether, 18 breeding attempts were followed in the three study years, with 33 nestlings checked for ticks. Ticks were collected from 20 nestlings (60.6%, *n* = 33, 72.2% of all nests had ticks), with six nestlings in four different nests in 2018, eight nestlings in five different nests in 2019 and six nestlings in four different nests in 2020. The distribution of nests visited and tick species recorded is presented in Fig. 1. Altogether 320 individual ticks were removed (314 identified to species level), belonging to five different species: *R. turanicus* was the most common species (mean prevalence 85.71%; mean intensity 16.44, CI 14.1–18.8). This species was present at all but one nest site in 2018, and was present at each site in 2019 and 2020. All *R. turanicus* individuals were adults, with a sex ratio of 7.45 in favor of males. *Hyalomma marginatum* (mean prevalence 20.0%; mean intensity 3.75, CI ± 4.9) was identified at three nesting locations (on four individual nestlings), with a total of three males, 11 nymphs and one larva collected. All the other three tick species were identified in single individuals (at three different locations), with one adult female of *Haemaphysalis erinacei* and *Ha. punctata* and one nymph of *Ixodes ricinus* collected (Table 1). Co-infection of two different tick species was established on three different nestlings, with one case each for *R. turanicus–Hy. marginatum*, *R. turanicus–Ha. punctata* and *R. turanicus–I. ricinus* species pairs.

**Table 1:** Tick species and developmental stage collected from Eurasian eagle owl (*Bubo bubo*) nestlings in Bulgaria, 2018–2020

Tick species	Development stage			
	Males	Females	Nymphs	Larvae
*Rhipicephalus turanicus*	261	35		
*Hyalomma marginatum*	3		11	1
*Haemaphysalis erinacei*		1		
*Haemaphysalis punctata*		1		
*Ixodes ricinus*			1	

**Fig. 2:**
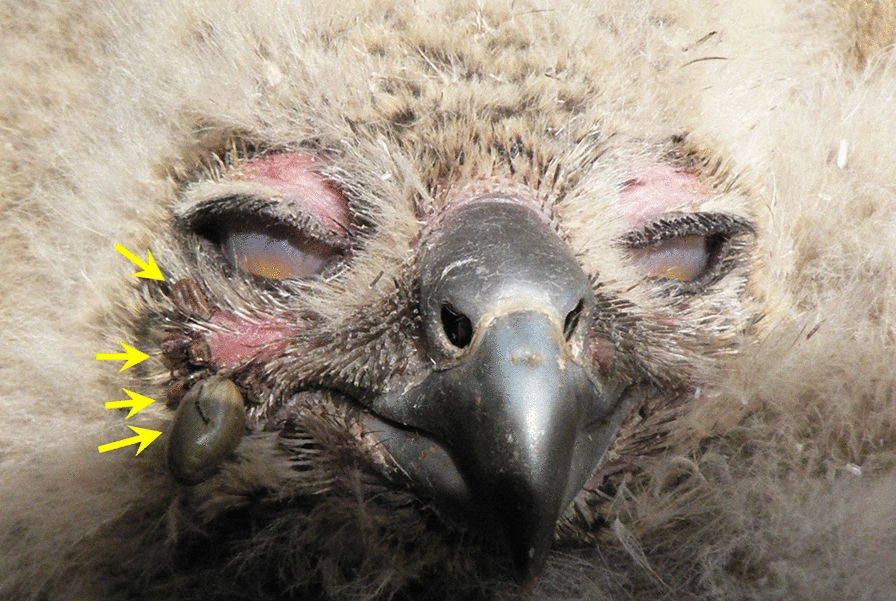
Engorged ticks on a Eurasian eagle owl (*Bubo bubo*) nestling in eastern Bulgaria

**Fig. 3:**
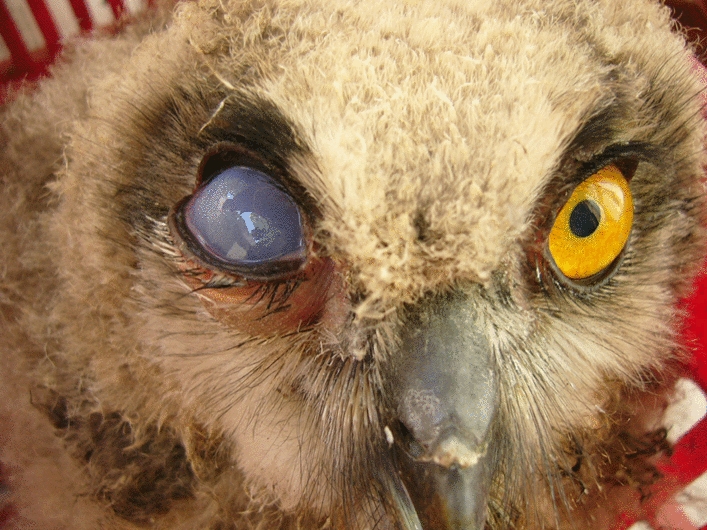
Eurasian eagle owl (*Bubo bubo*) nestling with keratitis (and also uveitis) developed on eye due to tick-induced inflammation

Ticks showed differential selection for host body parts, with most ticks being collected on the face and close to the beak (along the mandibles), neck and chin, eyelids and lore, and only a few individuals found in other regions of the body (Figs. 2, 3 and Table 2).

**Table 2:** Distribution of individual ticks on different regions of the body of Eurasian eagle owl (*Bubo bubo*) nestlings

Body part	No. of cases (no. of nestlings)	No. of ticks
Face and mandible	16	222
Eyelids	9	22
Lore	3	7
Chin	6	36
Forehead	1	4
Neck	2	26
Toes	2	2
Cloacal region	1	1

**Fig. 4:**
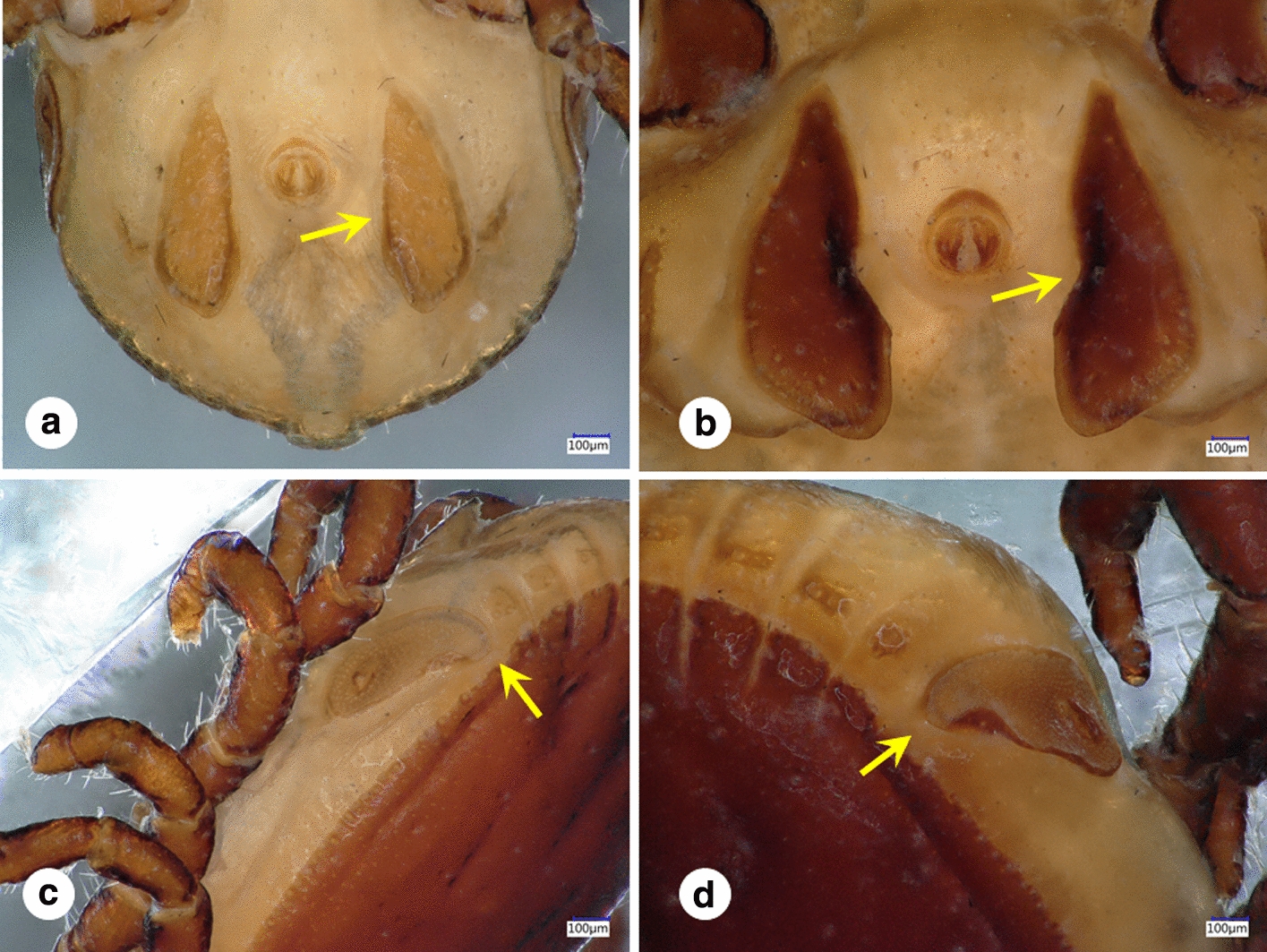
*Rhipicephalus turanicus* males with contrasting differences in adanal plates

**Fig. 5:**
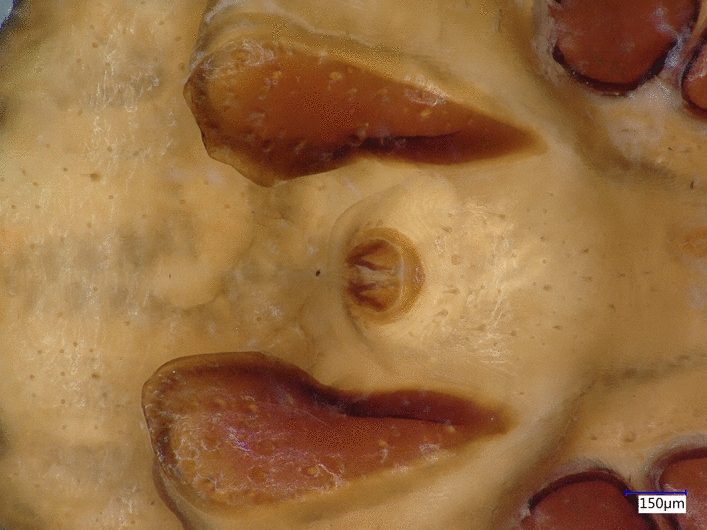
‘Intermediate type’ individual male *Rhipicephalus turanicus,* with asymmetrical adanal plates

*Rhipicephalus turanicus* males exhibited contrasting differences in morphology (Fig. 4), with two different morphotypes identified. Differences were noted in the length and width of idiosoma, coxae, adanal plates and scutum (Table 3), as well in the shape of the adanal plates (Fig. 2a, b) and spiraculae (Fig. 2c, d). Males belonging to the ‘larger’ morphotype (Fig. 2b, *n* = 214, 81.9%) showed adanal plates resembling the nominate form (see Fig. 131 in [38], while adanal plates of ‘small’ males (Fig. 2a, *n* = 44, 16.8%) resembled the adanal plates of *R. sanguineus* ‘eastern lineage’ (Fig. 1 in [35]) or the recently described *R. afranicus* (Fig. 10A in [34]). These differences were consistent in most males belonging to the two morphotypes; however, three individuals (1.1% of all males) showed an asymmetrical pattern, with visible differences between their respective left and right adanal plates (adanal plates of one such ‘intermediate-type’ male is shown in Fig. 5). Both morphotypes were present simultaneously at several nest sites (in 7 out of 13 cases), and thus they showed no contrasting geographical pattern. However, the ratio of ‘small’/‘large’ morphotypes was significantly larger in 2020 (33/132, 22.4%, *Χ*^2^ = 4.494, *p* < 0.05) than in 2018 (6.5%) or 2019 (5.7%).

**Table 3:** Morphological measurements of the two different morphotypes of male *Rhipicephalus turanicus* collected from nestling Eurasian eagle owls (*Bubo bubo*)

	Morphotype				t*
	‘Small’		‘Large’		
	Mean	SE	Mean	SE	
Idiosoma length	3275.92	48.71	4266.39	112.35	−8.089
Scutum length	2427.57	31.70	3035.82	63.45	−8.575
Scutum breadth	1582.19	33.41	2051.76	40.04	−9.005
Gnathosoma length	574.97	9.71	756.41	30.08	−5.739
Gnathosoma breadth	623.28	10.14	775.92	23.93	−5.873
Adanal plate length	706.01	18.26	996.60	36.41	−7.135
Adanal plate breadth	269.63	11.11	389.06	13.91	−6.708
Coxa II. length	370.23	7.29	492.74	21.42	−5.414
Coxa II. breadth	276.01	4.55	361.76	9.29	−8.288

All measurements are in μm, showing the mean and SE for the 10 individuals measured

*All tests two-tailed and significant at *p* < 0.001

**Fig. 6:**
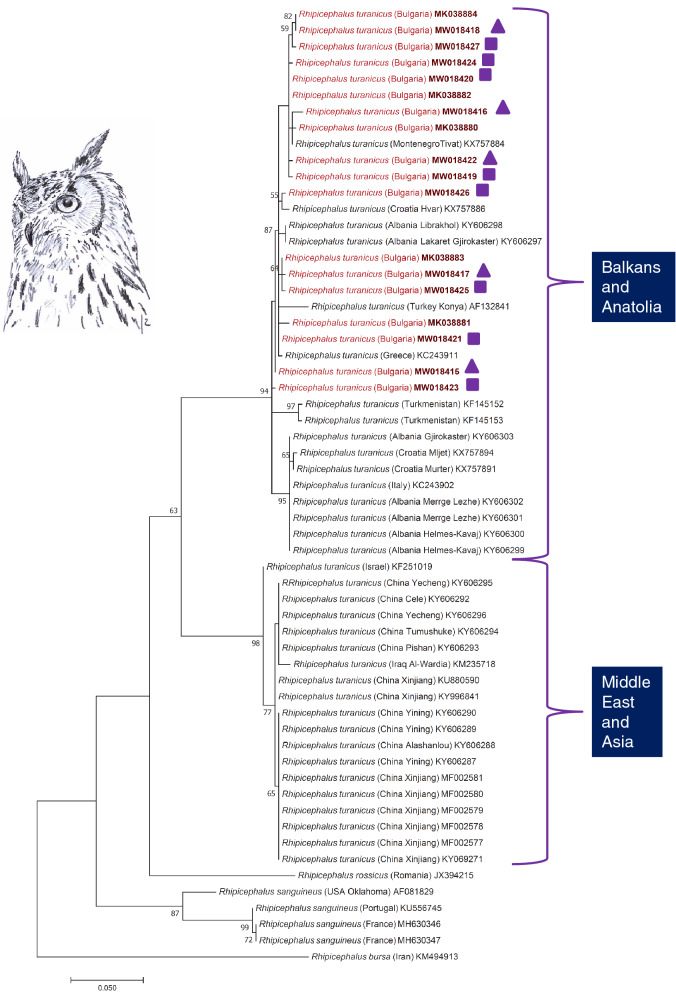
Phylogenetic comparison of *cox*1 sequences of *Rhipicephalus turanicus*. The genotypes of ticks collected in this study are marked with red. Branch lengths represent the number of substitutions per site inferred according to the scale shown (triangle ‘small’, square ‘large’ morphotype)

Concerning molecularly analysed ticks, all obtained *cox*1 sequences were most similar by BLAST analysis (99–100% identity) to *R. turanicus* sequences from Eastern Europe (KX757886) and Asia (AF132841). The 25 molecularly analysed ticks corresponded to 18 different *cox*1 haplotypes, with 619/630 to 628/630 bp (i.e. 98.3–99.7%) sequence identity inside the group. The representative haplotypes belonged to a phylogenetic group containing *R. turanicus* sequences from SE Europe, e.g. the Balkan Peninsula (Albania, Montenegro and Croatia) as well as Turkey (Konya). Different mitochondrial haplotypes were located even at the level of an individual eagle owl nest (Fig. 6, MK038880 and MK038881 were both collected in the same nest, although they differed in 11 substitutions, 619/630). No consistent geographical division among haplotypes was observed (ie. certain sequences with geographically close origins did not clustered together, see for example sequences from Croatia) within this group of *cox*1 sequences from Balkans and Anatolia which received relatively high support (94%). The node gathering the sequences of *R. turanicus* from China, Iraq or Israel and the ones from Balkans and Anatolia received low bootstrap value support (63%, Fig. 6). The mean sequence divergence within and between each of the two clades (Middle East-Asia clade and Balkans-Anatolia clade) was 0.0117 and 0.0559 respectively. We found no consistency in genetic differences between the two different morphotypes, as both 'small' and 'large' types did not clustered together in the same clade (Fig. 6).

All eagle owl nests contained multiple prey remains, with a mean number of 116.61 (95% CI 97–136) identifiable prey individuals across the different nest sites/years, with a diverse pattern of prey species identified. Altogether, 2103 individual prey items were identified, belonging to 122 prey species (data not shown). Identified prey remains showed a clear dominance of birds, but a diverse array of small to medium-sized mammals (rodents, insectivores and hares) were provided to each nest. Remains of suitable hosts for subadult stages of *R. turanicus* (small mammals: rodents, hedgehogs or hares [38]) were located in each nest, with small variations in numbers. We found a single correlation between diet (e.g. prey delivered to the nest) and the abundance of ticks on nestlings. A positive correlation (*r*_s_ = 0.430, *n* = 25, *p* < 0.05) was found between the total number of ticks and the number of medium-sized to large prey mammals (individual biomass > 100 g, e.g. individuals belonging to genera *Erinaceus*, *Lepus*, *Vulpes*, *Mustela*, *Felis*, *Spermophilus*, *Nannospalax*, *Arvicola* and *Glis*). No other relationship was found between the number of individual prey species or combinations of different prey categories (forest/grassland birds or mammals, rodents, etc.) and tick abundance or tick species composition in the respective nest.

Land use had an observable effect on tick parasitism of different nests. We found a significant effect of arable land cover, forest area and grasslands on the abundance of ticks. Our model showed that the most important predictor for tick abundance was the extent of arable land, while forests and grasslands contributed less, with no effect observed in the case of urbanized areas and watercourses (Table 4).

**Table 4:** Effect of land use on the presence and abundance of *Rhipicephalus turanicus* on nestling Eurasian eagle owls (*Bubo bubo*) (logistic GLMM)

Land use	Estimate	Standard error	Wald Stat	*p*
Intercept	−7.88099	0.546926	207.637	
Arable	12.97792	0.255392	2582.236	***
Forest	8.23005	0.934092	77.629	***
Grassland	5.12277	0.836783	37.479	***
Urban	0.00000			

Significance levels: ***p < 0.001. *GLMM* generalized linear mixed model

## Discussion

This is the first study on the tick infestation of the Eurasian eagle owl in Eastern Europe. We found high tick prevalence and a diverse tick assemblage (five tick species) on nestling owls. Three out of the five tick species were already reported from Eurasian eagle owls (*Hy. marginatum, I. ricinus* and *R. turanicus*), while for *Ha. erinacei* and *Ha. punctata* these are the first records. *Haemaphysalis erinacei* is a rare species in Europe, with only a handful of records from the region [39]. The spectrum of tick species recorded in Bulgaria partially overlaps with eagle owl-derived ticks collected in Portugal [9, 10] and Spain [25]. The diversity of tick species encountered on eagle owl nestlings was lower in comparison to the tick burden of adult birds in Portugal (where both *R. turanicus* and *Hy. marginatum* were collected, but with four additional species registered [9]). These differences might be related to host age (adult vs. nestlings) and regional differences, but also may be caused by reduced host defense (injured and/or weak individuals brought to animal rescue centers in the case of Western European studies). Interestingly, Silva et al. (2001) also reported only adults of *R. turanicus* from eagle owls. Reports of adults of *R. turanicus* from birds are scarce, with the only notable exceptions being birds from animal rescue centers in Portugal [9, 10] and anecdotal cases in Russia [30]. Both studies targeting breeding eagle owls—nestlings [25] or nest sites [22]—report only one tick species. Ortego and Espada [25] report only *Rhipicephalus* spp. from nestlings, considering its presence as ‘common’, but without finding any health-related impact on nestlings. Ticks found in several nest debris collections from eagle owl nest scrapes in Belgium contained only nymphs and larvae of the generalist tick *Ixodes ricinus*, with no information being provided about parasites on the nestlings themselves [22]. All these authors considered that the ticks were transferred to the nest sites with prey individuals provided by adults to the nestlings. Recently a cautionary note regarding the presence of *R. turanicus* in Portugal was raised [40], and the authors considered that the species is not present in Portugal, also suggesting that older records are results of misidentification.

In SE Bulgaria, the most common tick species found on nestlings was *R. turanicus*. It was present at most sites (17 out of 18) and was recorded at high intensity (mean intensity 16.44, CI 14.1–18.8). The collected individuals showed a wide range of morphological differences. Two morphotypes were found (‘small’ vs. ‘large’), which differed not only in the length/width of idiosoma, coxae, adanal plates and scutum (Table 3), but also in the form of the adanal plates (Fig. 4). Adanal plates are frequently used in identification of males belonging to the genus *Rhipicephalus* [31, 32, 34]. Here we report pronounced morphological differences in adanal plates of the two sympatric morphotypes of *R. turanicus* described here.

Morphological differences were so marked that species identity was ascertained by DNA extraction, followed by PCR and sequencing. Obtained sequences showed wide diversity (the 13 tested samples had five different haplotypes, with 98–99% similarity among one another, differing in up to 11 substitutions, 619/630), but all clustered with *R. turanicus* samples from the Balkans (Albania, Croatia and Montenegro) and Anatolia (Turkey). High morphological and molecular diversity was already reported for *R. turanicus* [41]; however, the geographic scale in the case of ticks collected from eagle owl nestlings is much smaller, as both forms and diverse sequences were found even at the level of the same eagle owl nest.

*Rhipicephalus turanicus* is a three-host tick species with a large distribution area (some regions of Africa and the hot arid or Mediterranean type regions of Eurasia), commonly reported in most countries with Mediterranean climate in Europe [38]. The species belongs to the *R. sanguineus* s.l. complex, which shows an intricate pattern of morphologically similar species distributed in the Mediterranean region [38, 42]. Based on the phylogenetic analysis presented here, *R. turanicus* shows a similar distribution pattern to *R. sanguineus* s.l.*,* with a parapatric occurrence of its haplotypes in the Mediterranean Basin [35]. Currently our knowledge is limited regarding the main driving force shaping these ranges, with geographic distribution of main hosts [43], local climatic adaptations [41] or glacial isolation and anthropogenic-resulting mixing [35] being suggested as possible causes. Regarding the present findings, such as morphological differences (the presence of ‘small’ and ‘large’ morphotypes) and high genetic diversity within sympatric populations of *R. turanicus* in SE Bulgaria, the most plausible explanation remains to be explored. High morphological variance is not unusual among the members of the *Rhipicephalus* genus; however, the sympatric occurrence of such a diverse assemblage of morphologically/genetically different forms is rare. We suppose that the sympatric occurrence of the two morphologically divergent forms (‘small’ and ‘large’) on eagle owl nestlings in Bulgaria may be the results of differences in climatic conditions and/or host species (source of blood meals for subadult stages) used during development, leading to size differences in the two lineages. Similar finds were already observed in the African *Rhipicephalus* species (*R. appendiculatus*, Zambia, [44]), although the differences noted there were seasonal and altitude-dependent, in contrast to our case, where there are no climate, season or landscape differences recorded. In addition, we would like to highlight our finding of the ‘mixed type’ adanal plates on several male *R. turanicus* individuals (Fig. 5). These findings suggest that for morphological identification of *Rhipicephalus* spp., one should consider a number of different characters, as the use of adanal plates alone may not be enough.

Small ungulates and carnivores are considered the main hosts of adult *R. turanicus* [30, 38]; however, adults were also recorded from birds, chiefly raptors, owls and crows. Nymphs and larvae are hosted primarily by small and medium-sized rodents (voles, mice, rats, jirds, gerbils and hamsters), hares and hedgehogs, and rarely smaller birds (larks) or lizards [30]. Our results show that each breeding pair provided their nestlings with considerable numbers of suitable hosts of pre-adult stages of *R. turanicus* as prey. At most nest sites the diet of nestlings was dominated by medium-sized rodents (*Arvicola amphibius*, *Rattus* spp.), hares (*Lepus europaeus*) and hedgehogs (*Erinaceus roumanicus*), all these species being recorded previously as suitable hosts for subadult stages of *R. turanicus*. Thus, we consider that the most probable way the ticks arrived at the nesting site may be by accidental transport (e.g. adult owls carried ticks attached to prey individuals).

In addition, an attempt to locate free *R. turanicus* individuals in the vegetation (using flagging) close to the nesting site failed in July 2019. Thus, the hypothesis of differential host use of subadults may be considered here, as ticks found in the eagle owls’ nests might have developed from subadult stages using a very diverse host palette, with at least 22 mammal and 78 bird species being located as regular prey brought to the nest sites. While this hypothesis may provide arguments for the size differences found between the two morphotypes (Table 3), it does not yield an explanation for structural differences in spiracles or adanal plates (Figs. 4 and 5).

Modelling land use in the neighborhood of eagle owl nest sites provided interesting results as well, with arable land being the most important negative predictor of tick abundance, while the extent of compact areas of forest or grassland also negatively influenced tick abundance. This is not surprising, as tick occurrence is low on arable lands (due to disturbance and lack of suitable hosts) and grasslands (the grasslands in the study area are short-grass steppes), coupled with the fact that eagle owls avoid hunting inside forests. As a consequence, the highest tick abundance was recorded in areas with a complex microhabitat pattern (shrubbery, ecotone, diverse mosaic pattern) and reduced percent of arable land, habitats with a high chance of diverse mammalian fauna, which may host diverse tick species.

Eagle owl nestlings hosted a diverse and abundant tick fauna, with *R. turanicus* being the dominant tick species in SE Bulgaria. The presence and high intensity of *R. turanicus* adults on eagle owls is interesting and highlights the importance of ground-nesting birds for maintaining populations of ticks usually found on medium-sized mammals, with nestling owls offering an alternative host source for ticks otherwise failing to locate suitable hosts.

## Supplementary Information

**Additional file 1: Table S1**. Estimates of evolutionary divergence between sequences using the number of base differences per site. The analysis involved 40 nucleotide sequences, with codon positions including 1st + 2nd + 3rd + noncoding. All positions containing gaps and missing data were eliminated, with a total of 421 positions in the final dataset.

## Data Availability

All data generated during this study are included in this published article.
